# Bilateral glenohumeral internal rotation deficit (GIRD) in elite gymnasts

**DOI:** 10.1007/s00402-022-04577-0

**Published:** 2022-08-18

**Authors:** Ralf J Doyscher, Leopold Rühl, Benjamin Czichy, Konrad Neumann, Timm Denecke, Bernd Wolfarth, Scott A Rodeo, Markus Scheibel

**Affiliations:** 1 Center for Musculoskeletal Surgery and Department of Sports Medicine, Charité – Universitaetsmedizin Berlin, Augustenburger Platz 1, Berlin, Germany; 2 Institute for Biometry and Clinical Epidemiology, Charité - Universitaetsmedizin Berlin, Berlin, Germany; 3 Clinic for Radiology, Charité – Universitaetsmedizin Berlin, Berlin, Germany; 4 Sports Medicine & Shoulder Service, Hospital for Special Surgery, New York, NY USA; 5 Department of Shoulder and Elbow Surgery, Schulthess Clinic Zurich, Zurich, Switzerland; 6 Clinic for Diagnostic and Interventional Radiology, University Hospital Leipzig, Leipzig, Germany; 7 Borussia VFL 1900 Moenchengladbach GmbH, Moenchengladbach, Germany

**Keywords:** Gymnasts, GIRD, Capsular thickening, Humeral retrotorsion, Periscapular muscle hypertrophy

## Abstract

**Introduction:**

The “Glenohumeral Internal Rotation Deficit (GIRD)” is known as the difference in internal rotation range of motion (IRRM) between the dominant and non-dominant shoulder of overhead athletes as a result of asymmetric loading. As in contrast loading pattern in gymnastics are quite symmetric and structural changes often occur bilaterally, the question arises if GIRD might develop bilaterally in gymnasts as one source of common bilateral shoulder pathologies and to search for underlying structural adaptations.

**Materials and methods:**

A group of 35 elite gymnasts (8–24 years) were recruited from a local Olympic Training Centre and compared to a paired cohort of 28 non-overhead athletes. Clinical examinations, digital range of motion (ROM)-measurement, ultrasonographic humeral torsion measurement, and standardized MRI scans of both shoulders were obtained and examined for structural pathologies, cross-sectional areas (CSA) of the rotator cuff muscles and capsular thickness.

**Results:**

ROM-measurements showed significant decrease in IRRM in the gymnasts groups by age, with IRRM of 48.6° (SD: 8.4°, CI 95%: 43.0–54.3°) at age group 1 (8–10 years) and IRRM of 10° (SD: 11.4°; CI 95%: 0–22.0°) at age group 4 (18–26 years), that was statistically significant for the entire cohort (*p* = 0.017) compared to the controls. CSA were not significantly different between the cohorts, while there was a slightly increased humeral retrotorsion in the gymnasts as well as a statistically significant posterior capsular thickening.

**Conclusion:**

A new bilateral form of GIRD was identified in higher age groups of youth and senior elite gymnasts enrolled in this study. Despite to former definition of GIRD there was no compensatory increase in external rotation range of motion (ERRM) but an association with posterior capsular thickening, while there was no periscapular muscle hypertrophy. Humeral retrotorsion was also slightly increased in the gymnasts group.

## Introduction

In overhead sports such as gymnastics the shoulder joint is still a main focus of orthopaedic problems. As acute injuries only contribute to a minor part of all shoulder-related problems, overuse pathologies remain the most common reasons for medical treatment [2, 13, 17]. Some of these overuse conditions are linked to sport-specific structural adaptations, e.g. thickening of tendons, muscles, and connective tissue provoked by repetitive movements and minor injuries [3, 19, 21]. One of these adaptations, the so called “Glenohumeral Internal Rotation Deficit Syndrome (GIRD)” was identified as a specific risk factor in the development of many shoulder injuries and overuse pathologies [19]. There is strong evidence that GIRD is involved in the development of many overuse injury patterns in overhead athletes including SLAP lesions, labrum tears, subacromial bursitis and structural lesions of the rotator cuff [12, 20, 24].

Previously GIRD was defined as the difference in internal rotation range of motion (IRRM) between the dominant and non-dominant limbs of an athlete [8].

In contrast to other sports, most of the gymnastic exercises demand a bilaterally and symmetric loading of both shoulders [16]. Therefore, the question arises if similar changes of the shoulder structures might occur bilaterally in this athlete population in contrast to the already known unilateral GIRD that usually affects only the dominant side, as well as how these changes develop during the maturation of young adolescent elite gymnasts.

## Methods

### Participants

A group of 35 high-level gymnasts aged between 8 and 24 years (average 13.1 years) were recruited from the local Olympic Training Centre. These athletes were matched to a control group of 28 non-overhead athletes. For evaluation, the athletes of both cohorts were separated into four age groups according to the gymnasts training levels as follows: group 1: 8–10 years (*n* = 10), group 2: 11–13 years (*n* = 12), group 3: 14–16 years (*n* = 4), and group 4: 18–24 years (*n* = 6) for the gymnast cohort and group 1: 8–10 (*n* = 9), group 2: 11–13 years (*n* = 8), group 3: 14–16 years (*n* = 4), and group 4: 18–24 years (*n* = 6) for the control cohort. The average exercise-time per week in the gymnasts group reached between 20 h in group 1 and 35 h in group 3 and 4 depending on the age of the athletes. All gymnasts had already reached an elite level and participated in all sports-specific exercises and figures.

### Inclusion and exclusion criteria

The participants were matched according to sex, age, height, and weight and side dominance. The matched-pair inclusion criteria were limited to an individual difference in height of 5% as well as 15% for weight. Further inclusion criteria were a regular sport activity of at least 8 h per week and the ability of full sport participation at the time of enrollment and examination.

Exclusion criteria were: acute traumatic injuries of one or both shoulders within 3 months prior to enrollment, recent surgery within the 6 month prior to enrollment, disability to full participating in the sports activity due to any reason at the moment of enrollment and examination as well as 4 weeks prior to it.

### Ethical approval

The study protocol was approved by the central ethical review committee of the local university. Informed consent was obtained from all study participants and parents (for those under the age of 18 years) before enrolling into the study.

### Clinical examination

All athletes completed a standardized questionnaire and underwent a clinical examination as well as an isometric strength measurement to evaluate for the Constant score, the subjective shoulder value and to test for shoulder pathologies. All examinations were conducted by two independent and clinically experienced examiners (the first and the senior author). The clinical tests included examinations for instability, rotator cuff tears, subacromial bursitis, SLAP-, and labrum lesions.

A digital inclinometer (The Saunders Group Inc. Chaska, Minnesota, USA) was used to perform the range of motion measurements. The evaluation included measurements for shoulder abduction and adduction, elevation, as well as internal and external rotation in neutral position and at 90° shoulder abduction. To evaluate for high internal and external rotation range of motion participants were placed supine on the treatment table with 90° of shoulder abduction and elbow flexion. Scapular stabilization was provided by the examiner through a posteriorly directed force at the acromion fixating the scapula at the surface of the treatment table to isolate glenohumeral joint motion.

### Radiological examination

**Fig. 1:**
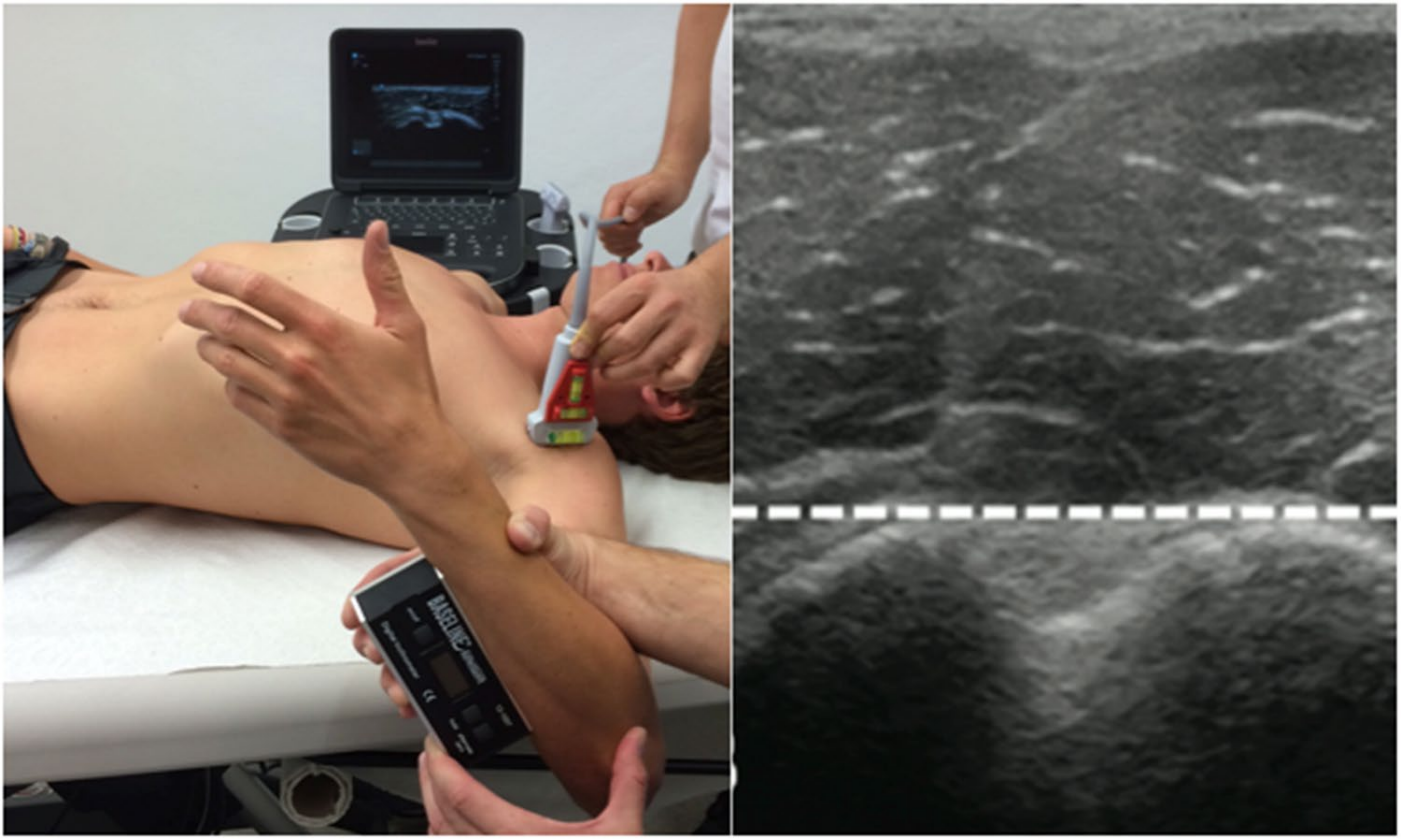
Left: Technique of indirect sonographic assessment of humeral torsion. Right: Ultrasonographic image of the proximal humerus with the apices of the humeral tubercles aligned horizontally

Humeral torsion was assessed utilizing the indirect ultrasonographic technique described by Myers et al. [14], Whiteley et al. [23], and Yamamoto et al. [25]. Participants were placed supine on a treatment bench with their shoulders at 90° abduction and elbow flexion. A tester positioned a 6 cm linear array ultrasound transducer (Edge I, SonoSite, Inc. FUJIFILM, Bothell, USA) anteriorly on the subject’s shoulder with the ultrasound transducer parallel to the level of the treatment table (verified with a custom-made bubble level attached to the ultrasound transducer) and aligned perpendicular to the long axis of the humerus in the frontal plane. The second examiner rotated the humerus until the bicipital groove appeared in the center of the ultrasound image, with the line connecting the apices of the greater and lesser tubercles parallel to the horizontal plane. The second examiner then placed a digital inclinometer on the ulnar side of the forearm, pressing firmly against the ulna, and recorded the forearm’s inclination angle with respect to the horizontal line (Fig. 1).

Because the ulna extends perpendicular to the elbow’s epicondylar axis (line connecting the medial and lateral epicondyles), this angle reflects the angular difference between the epicondylar axis (distal humerus) and the line perpendicular to that connecting the apices of the greater and lesser tubercles (proximal humerus), thus representing an ultrasound-assisted approximation of humeral retrotorsion [8].

A series of 1.5T MRI scans were performed on both shoulders in all 63 subjects (126 shoulders). The MRI protocol was adapted according to De Carli et al. including Axial T2, Axial TSE T1, Coronal T1, Coronal T1 TSE, Coronal TIRM, and Sagittal TSE T1 scans [4, 5]. Subjects were placed supine with the arm at the side in internal rotation with elbow flexion, as described by Davis et al.[4]. The MRI scans were evaluated for structural pathologies by two independent radiologists and an experienced senior trauma surgeon specialized in arthroscopic surgery of the shoulder (the senior author). In addition the cross-sectional areas of the supraspinatus, the infraspinatus, the subscapularis and the teres minor muscle were measured in the well-defined y-plane of the Sagittal TSE T1 sequence, as well as of the deltoid muscle in the Axial T1 sequence at the level of the maximal diameter of the humeral head. The thickness of the articular capsule of the glenohumeral joint was measured at 4 points: anterior and posterior at the level of maximal diameter of the humeral head in the Axial TSE T1 sequence anterior at 12 o`clock position and posterior at its origin point at the glenoid labrum according to the ultrasonographic studies of Thomas et al. [21]. The cranial capsule diameter was measured at the highest point of the capsule in the Axial TSE T1 sequence showing the maximal diameter of the humeral head and the caudal diameter at the lowest point of the caudal recessus. MRI and sonographic studies were all independently evaluated by a specialized radiologist and the two clinical examiners.

### Statistical methods

For all metrical variables means, standard deviations (SD) and two-sided 95% confidence intervals for all age groups and for the test and control group were calculated.

Comparisons of the gymnasts with the control subjects in the same age group were performed using the *t* test for paired samples whereas for comparisons of all gymnasts with their controls the non-parametric sign test for paired samples was applied. The level of significance was *α* = 0.05. All statistical analyses were carried out using the software R 3.1.0 for statistical computing.

## Results

### Clinical examination and tests

The results of the clinical examination and the questionnaire showed normal Constant score values for the gymnasts group of 85.5 points for the right side and of 85.3 points for the left side as well as for the control group with 88.7 points for the right side and 89.2 points for the left side. SSV-Score values reached 93.5% for the right side and 91.9% on the left side in the gymnasts cohort compared to 98.3% for the right side and 99.2% for the left side in the controls. The results of the LHB score showed age-related normal values of 96.5 right and 96.2 left for the gymnasts and 94.1 right and 93.8 left for the control athletes.

None of the athletes reported pain or shoulder-related complaints at the time of the examination, while 5 of the 6 gymnasts aged older than 18 years had already undergone arthroscopic surgery of at least one shoulder during the prior course of their career. No shoulder-related medical treatment was noted in all athletes of the control cohort. It is known that many, especially experienced elite gymnasts are constantly competing with ongoing discomfort or even pain in their shoulder joints. Major injuries and need for arthroscopic treatment in higher age groups are very common. Therefore, it was within the specific interest and aim of this study to compare their functional and structural condition with a non-overhead sports group representing a more or less physiological, non-impaired control group.

### Range of motion measurements

In age group 1 (8–10 years), 1 of 11 athletes (9%) admitted to have experienced shoulder problems that required medical treatment other than surgery, in age group 2 there were 30% (*n* = 4) and 60% in group 3 (*n* = 3) as well as 83% in group 4 (*n* = 5). No shoulder-related medical treatment was noted in all athletes of the control cohort including medical consultancy, physiotherapy, need for medication or surgery.

**Fig. 2:**
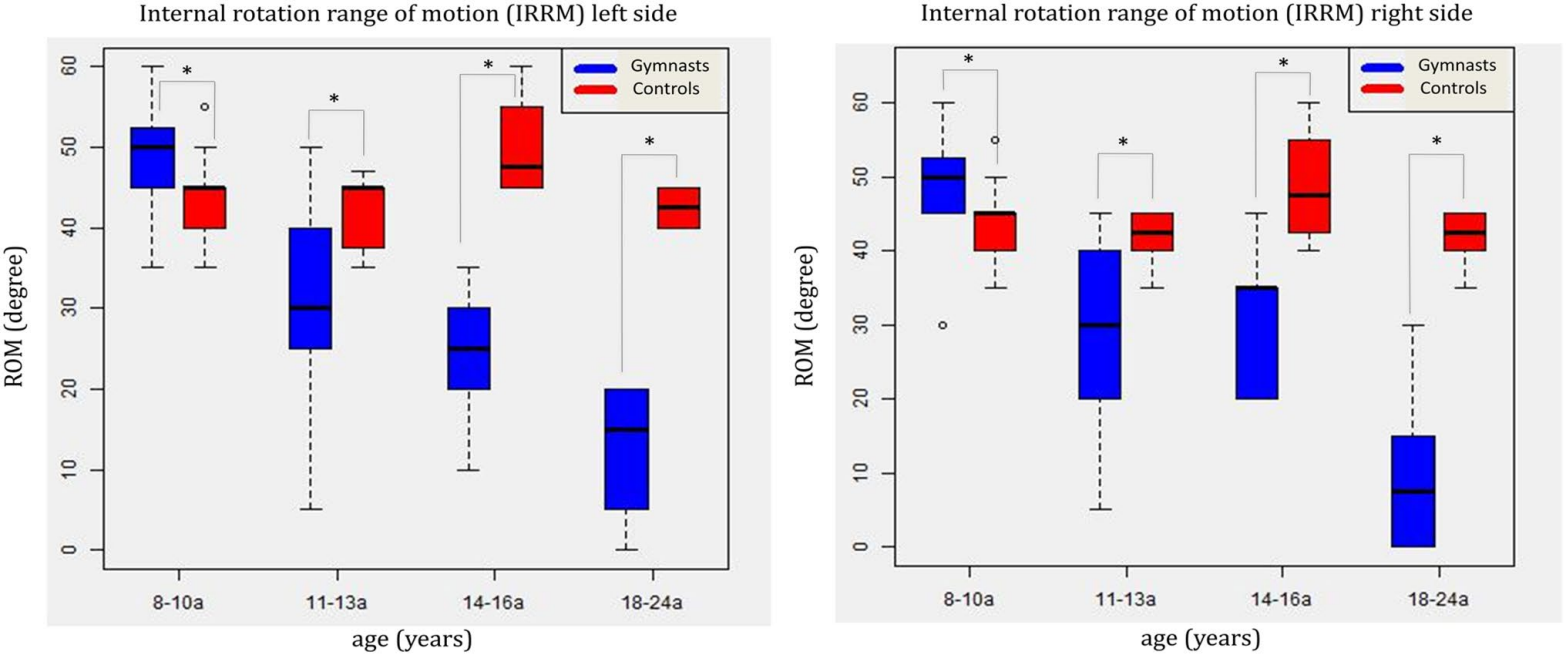
Comparison of the internal rotation range of motion of the gymnast and the control cohort showing the development of GIRD in the gymnast groups (*P* = 0.017 (right) and P = 0.009 (left))

The measurement of shoulder ROM showed a decrease of internal rotation range of motion at 90° abduction position (IRRM) in the gymnast groups. This decrease in IRRM was symmetric for both sides and was found to be directly proportional to the gymnast’s age. The average values of IRRM started for the right side with 48.6° (SD: 8.4°, CI 95%: 43.0–54.3°) in age group 1 as well as of 48.6° (SD: 8.4°, CI 95%: 43.0–54.3°) for the left side. 29.2° (SD: 12.0°, CI 95%: 21.9–36.5°) right and 30.0° (SD: 12.4°, CI 95%: 22.5–37.5°) left side in group 2, as well as left side 31.0° (SD: 10.8°, CI 95%: 17.5–44.5°) and right side 24.0° (SD: 9.6°, CI 95%: 12.1–35.9°) and ended at a mean IRRM of 10° (SD: 11.4°; CI 95%: 0–22.0°) right side and 12.5° (SD: 8.2°, CI 95%: 3.9–21.1°) at age group 4 (Fig. 2).

**Fig. 3:**
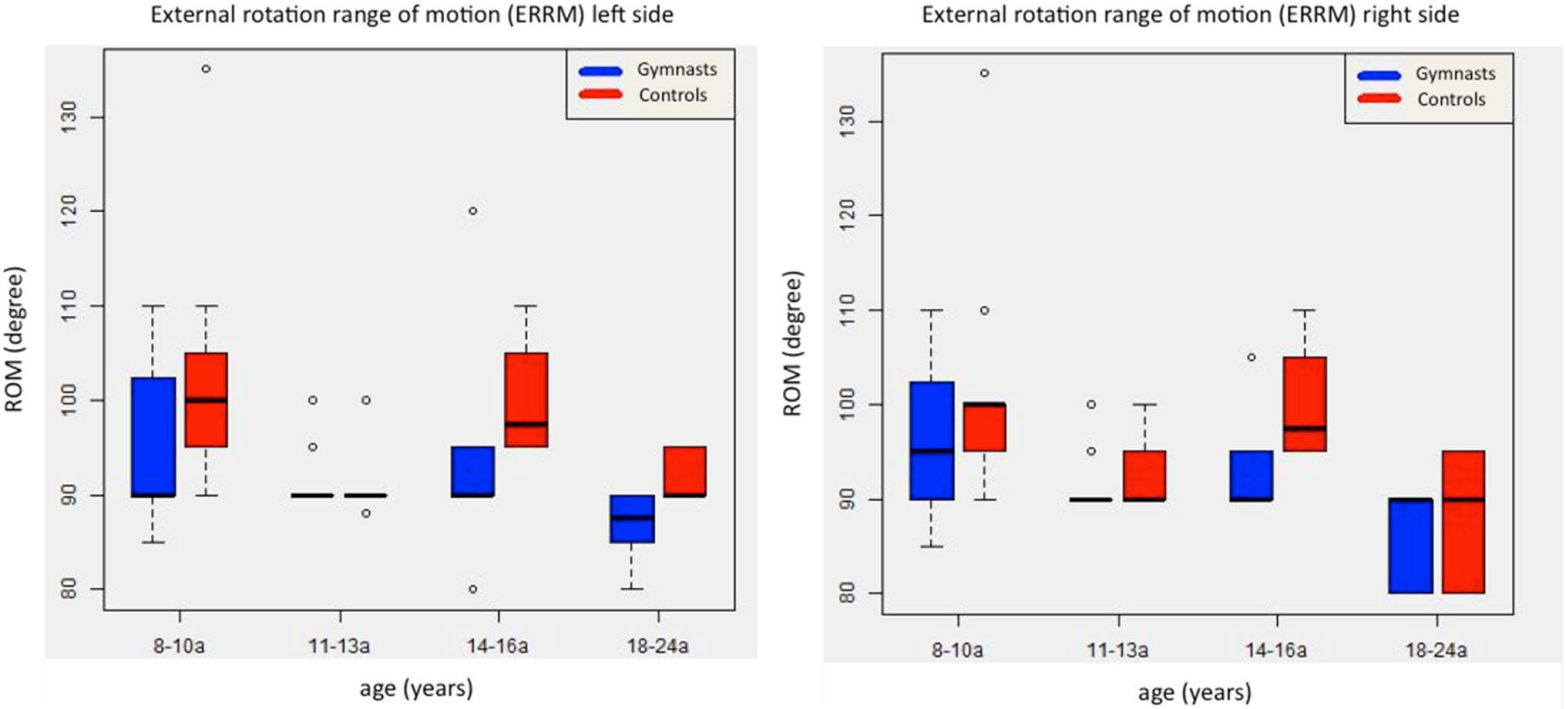
Comparison of external rotation range of motion (ERRM) between gymnasts a control cohort. In contrast to the decrease of IRRM the ERRM remains in a physiological range through all age groups

The IRRM in the matched controls remained in a normal range throughout all age groups with an average for the right side of 44.4° (SD: 7.0°, CI 95%: 41.7–47.2°) and 44.7° (SD: 6.4°, CI 95%: 42.2–47.2°) for the left side. The difference between the gymnast and the control group was statistically significant regarding the entire cohorts (right side: *p* = 0.017, left side: *p* = 0.009) as well as in comparison for specific age groups (Figs. 2, 3).

**Fig. 4:**
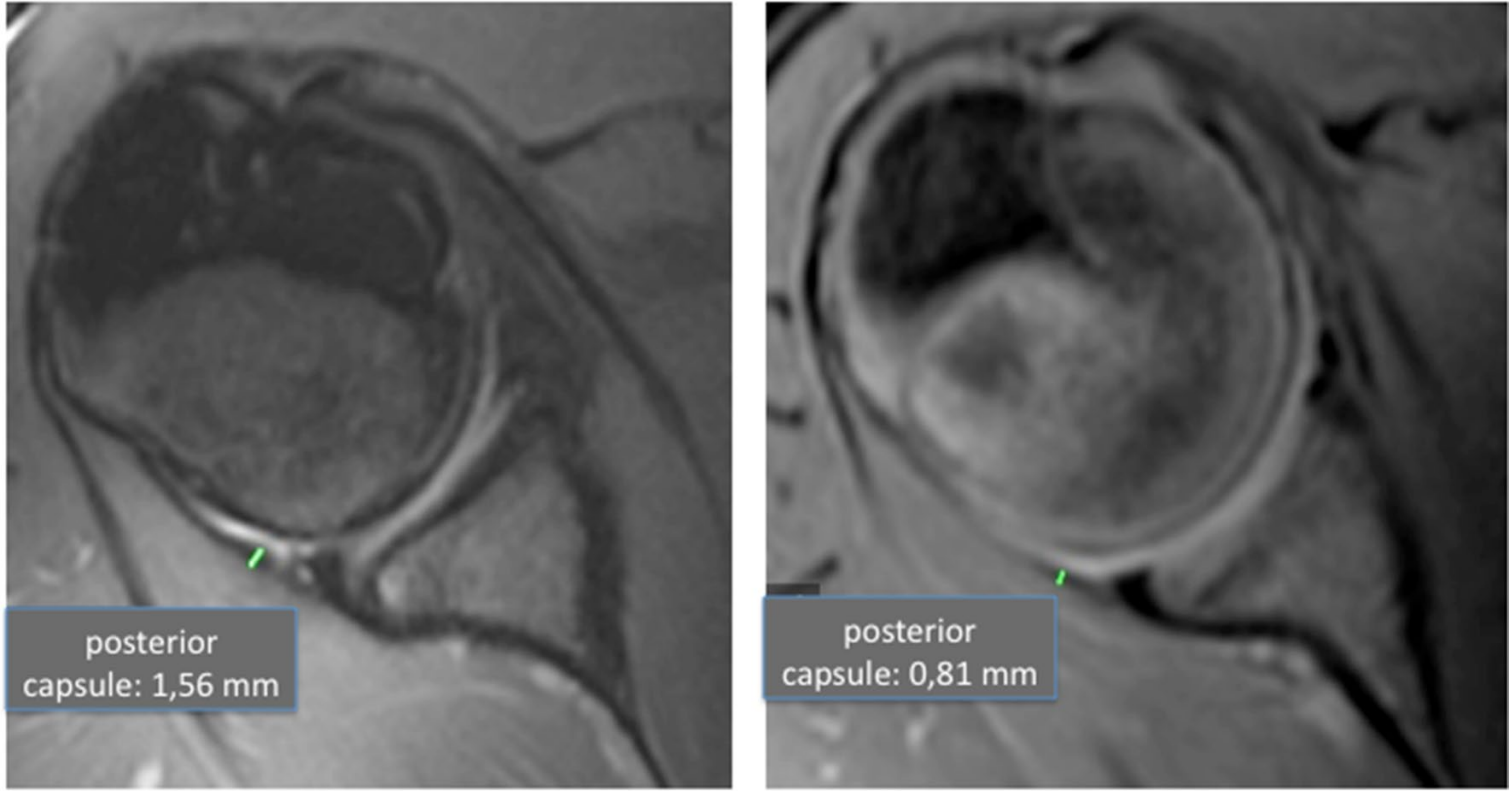
Left side: example of an Axial T1 Sequence of a rather thick posterior capsule of a 12-year-old gymnast; right side: according matched control

The external range of motion (ERRM) remained nearly consistent for both cohorts and within all age groups with a mean of 92.4° (SD: 6.8; CI 95%: 90.1–94.8°) in gymnasts and a mean of 95.9° (SD: 10.4; CI 95%: 91.8–100.0°) in the control group (*p* = 0.021) (Fig. 4).

### Radiological results

The bilateral MRI scans were examined for structural pathologies and sport-specific changes by two independent radiologists and one experienced orthopaedic surgeon. No major pathologies were found except 2 degenerative labral defects and 3 minor partial rotator cuff tears in the gymnasts group 4. These findings have been estimated as being minor conditions and without any clinical relevance or need for specific treatment at the time of the examination as there was no correlation to actual complaints or positive clinical tests. Gymnasts with degenerative changes in the MRI were not excluded from the study as it was aimed to compare a cross-sectional evaluation of the shoulders of active gymnasts to a non-overhead group.

The muscle cross-sectional areas (CSA) of the examined muscles showed a physiological increase with regard to the corresponding age, with a slight tendency towards greater CSA in the gymnast cohort but no significant differences between the groups (Table 1).

**Table 1:** Comparison of the cross-sectional areas (cm^2^) of the rotator cuff muscles between gymnasts and control group, *SSP* supraspinatus, *ISP* infraspinatus muscle, *SSC* subscapularis muscle

Group	SSP right	SSP left	ISP right	ISP left	SSC right	SSC left	Teres minor right	Teres minor left
Gymnasts (total. *n* = 32)	5.7 (SD 2.1; CI 95%: 5.0–6.5)	5.7 (SD 2.0; CI 95%: 5.0–6.4)	19.4 (SD 8.3; CI 95%: 16.4–22.5)	19.0 (SD 8.4; CI 95%: 15.9–22.0)	20.9 (SD 6.0; CI 95%: 18.6–23.2)	20.5 (SD 6.8; CI 95%: 18.1–23.0)	23.6 (SD 9.3; CI 95%: 20.2–27.0)	23.7 (SD 9.4; CI 95%: 20.3–27.1)
Control (total. n = 27)	6.1 (SD 2.8; CI 95%: 5.0–7.2)	6.0 (SD 2.8; CI 95%: 4.9–7.1)	20.6 (SD 8.1; CI 95%: 17.4–23.8)	20.5 (SD 8.3; CI 95%: 17.2–23.7)	22.6 (SD 7.8; CI 95%: 19.4–25.8)	21.4 (SD 7.2; CI 95%: 18.6–24.3)	25.2 (SD 9.2; CI 95%: 21.6–28.9)	24.5 (SD 9.8; CI 95%: 20.6–28.4)
Gymnasts–Control	− 0.2 (SD 1.7; CI 95%:− 0.9–0.4); *p* = 1.000	− 0.2 (SD 1.5; CI 95%:–0.8–0.4); *p* = 1.000	-0.6 (SD 4.4; CI 95%:− 2.3–1.2); *p* = 0.845	− 0.7 (SD 4.2; CI 95%:− 2.4–1.0); *p* = 0.845	− 0.8 (SD 5.1; CI 95%:− 2.9–1.3); *p* = 1.000	− 0.4 (SD 4.7; CI 95%:− 2.3–1.5); *p* = 0.541	− 0.9 (SD 4.8; CI 95%:− 2.9–1.0); *p* = 0.845	− 0.0 (SD 4.9; CI 95%:− 1.9–2.0); *p* = 0.701

The measurement of the capsule thickness regarding the anterior, superior und inferior capsule revealed similar capsule diameter values in both cohorts with respect to the compared age groups.

**Fig. 5:**
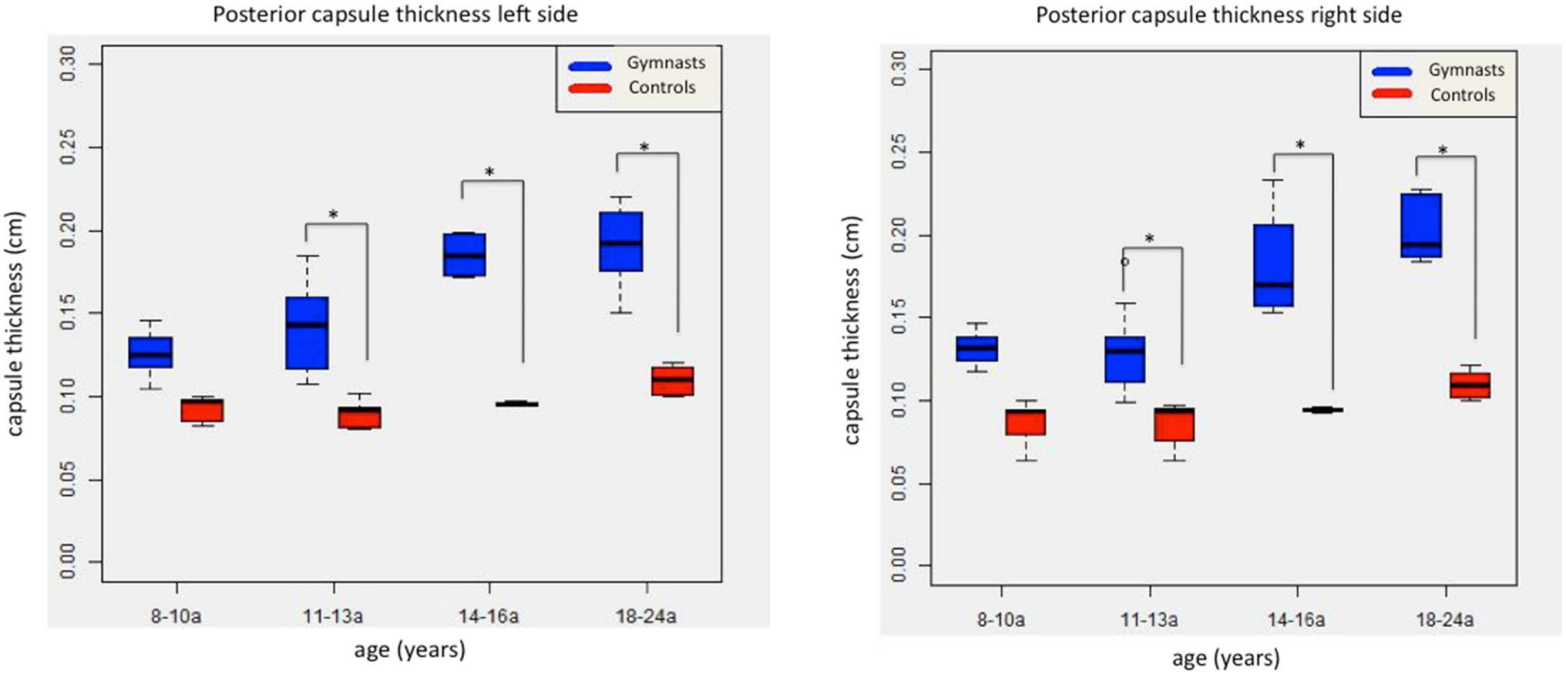
Development of the posterior capsule thickness of the left and right side compared of the age groups of the gymnast and control cohort (*p* < 0.001)

However, the thickness of the posterior capsule was found to increase between the gymnast groups according to age and weight, reaching from a mean thickness of 0.13 cm (SD: 0.01, CI 95%: 0.13–0.14 cm) in group 1 up to 0.2 cm (SD: 0.02; CI 95%: 0.18–0.22 cm) in group 4. In comparison, the posterior capsule thickness in the controls showed less of an increase, with a mean of 0.09 cm (SD 0.01; CI95% 0.08–0.10 cm) in group 1 up to a mean thickness of 0.11 cm (SD: 0.01; CI 95%: 0.10–0.12 cm) in group 4. The differences between the gymnasts and the controls was statistically significant (*p* < 0.001, Fig. 5).

The ultrasonographic torsion measurement of the gymnasts group revealed forearm inclination angles with a mean of 82.86° (SD: 4.6) right and 83.08° (SD: 5.2) left side, resulting in humeral retrotorsion angles (HRT) with a mean of 7.14° (SD: 1.2) for the right side and a mean of 6.92° (SD: 1.4) for the left side. Side-to-side differences were less than a mean of 1.4°. There was a tendency towards lower torsion values in the older age groups (group 1: 12.72°, group 2: 7.4° group 3: 6.1°; group 4: 5.8°).

## Discussion

### Recent concepts of the aetiology of GIRD

Early concepts of the aetiology of GIRD published by Burkhart et al. and others claimed that repetitive eccentric loading of the shoulder during the throwing or striking movement will lead to an over-stretch of the anterior structures while there will be a compression mechanism of the dorsal capsule, leading to chronic irritation with thickening of the dorsal capsule and an over-stretch of the anterior ligaments. The result is shifting of the center of rotation of the shoulder towards a more cranial and posterior position [3, 20, 21].

This might lead to a change of biomechanical force distribution and force direction and might affect the loading of the rotator cuff and the anterior labrum as well as a forced torsion loading of the LHB tendon and the SLAP-complex [2]. While there is an ongoing discussion whether soft tissue changes or bony adaptations are the primary contributors to the development of GIRD [8, 20] recent studies widely agree that thickening of the posterior shoulder joint capsule and increased humeral retrotorsion can be considered as the two identified main factors [22, 24].

### Structural and functional changes of the Gymnasts shoulder

In modern gymnastics, the athlete`s shoulder is also exposed to enormous eccentric and concentric forces and exercises even contain biomechanic sequences comparable to the course, velocity and force distribution of the striking movement [1, 16]. Furthermore the same overuse injury pattern, e.g. rotator cuff tears, SLAP lesions and labrum degeneration are reported in up to 100% of the experienced high-level athletes in this sport [5].

In contrast to former data presented by DeCarli et al. who described pathological lesions of the shoulder in up to 100% of the examined elite gymnasts we only found minor pathologies in the adult gymnast group at the time of the evaluation. However, five out of six athletes older than 18 years had already undergone arthroscopic treatment of one or both shoulders prior to the study [5]. This might point out that overuse injuries in this sport mainly occur in higher age groups and were most probably induced by long standing overuse and repetitive injuries [1]. As there was a correlation of increase in GIRD with age and the occurrence of shoulder problems and as the decrease in internal range of motion was already demonstrated in younger gymnasts without these problems, it seems possible that GIRD has influenced the emergence of these problems or even might have been a precursor of them.

All clinical scores used to determine the over-all general status of the shoulder function and well-being of the participants were within the age-related normal range, showing that the athletes were healthy and without any restriction for participation in the study.

### Description and definition of GIRD

Previously the GIRD Syndrome was described as a “decrease of internal rotation range of motion of the dominant limbs compared to the non-dominant side” [21, 24]. This definition implies that the grade of decrease of the internal rotation ability is usually assessed by side-to-side comparison within the same individual, and the cut-off values for defining decreased values as GIRD were taken individually; e.g. more than 25° side-difference in IRRM [9]. As we expected to find bilateral and more or less symmetric changes in our gymnast cohort, we felt that a comparison with a non-overhead athlete group was necessary. Although we were not able to define GIRD by comparing the dominant to non-dominant side, we instead defined an absolute cut-off in comparison of gymnasts and their matched controls. We found an almost linear decrease in the humeral internal rotation ability in the gymnasts with age. Between the age of 12 and 14 years (group 2 and group 3) the IRRM fell below 25° of internal rotation. It can be suggested that by this point clinical GIRD occurs in these athletes as also the number of conditions requiring medical treatment of the shoulder increases [8].

The findings of this study indicate that there is a bilateral form of GIRD that develops in the examined gymnast cohort. It occurs symmetrically on both sides. Current understanding of GIRD suggests that structural changes occur in response to long standing and repetitive loading, leading to a decrease in internal rotation range of motion with a simultaneous increase in external rotation range of motion [11, 18, 19, 21]. This means that the total rotation range of motion remains constant. However, the bilateral form of GIRD that was found in our study cohort did not show any increase in external rotation range of motion, resulting in a slight decrease of the total rotation range of motion.

### Association to posterior capsule thickening and humeral torsion

Thickening of the posterior capsule and increased humeral retrotorsion have been identified as the main etiological reasons for the development of GIRD, The results of humeral retrotorsion measurements in overhead athletes has led to controversy as to whether humeral torsion or changes in soft tissue structures (capsule) predominantly contribute to GIRD in overhead athletes [8]. The data of this study also clearly show a strong association between posterior capsule thickening (PCT) and the development of GIRD, as it was also reported by Tehranzadeh et al. and others [15, 20, 21]. There was no significant correlation to hypertrophy of the periscapular muscles. This might point towards a different aetiology and pathomechanism due to the different sport-specific loading pattern in the gymnastic sport that normally impact bilaterally and requires extreme external rotation movements less often than throwing sports like baseball [7, 8, 10, 20, 25].

During the aging process there is a natural change in the physical properties of connective tissue resulting in increased stiffness of muscles and tendons [8]. In the adolescent throwing athlete, this process of collagen turnover occurs concomitant with the development of humeral retrotorsion, and each may exert different influences on shoulder ROM during the maturation process. Therefore, it has been postulated that the soft tissue surrounding the shoulder of young athletes may provide less constraint or influence on shoulder mobility compared to bony adaptions [7].

The values of humeral retrotorsion that were found in the gymnasts group in this study with values of 7.14° (SD: 1.2) right and 6.92° (SD: 1.4) left side do not appear significantly increased on the first view as the generally accepted “normal” value for adult retrotorsion is between 25° and 35° [6, 7]. Unfortunately it was not possible to perform the retrotorsion measurement in the complete control group due to organizational difficulties. The degree of humeral retrotorsion varies depending on age, sex, limb dominance, and race. Early examinations by Edelson et al. showed that at birth, the humeral head is in marked retrotorsion and undergoes a process of derotation (less retrotorsion) during childhood and adolescent years starting with a retrotorsion of about 65° at an age of 4 years up to a mean of 25° in adolescent and adult age [6]. Earlier studies reported humeral retrotorsion between 45° and 20° in other overhead athletes of the same age groups as in this study [7, 9, 25]. Most of these values have been obtained using CT scans while more recent studies were also using indirect ultrasound methods as in our examination and it must be emphasized that each method is measuring a different anatomic angle; thus, the data obtained from each method have to be compared with caution [10]. As prior studies indicate that bicipital-forearm angles and forearm inclination angles of greater than 70°are associated with a decrease in IRRM and, therefore, were considered as an increase in humeral retrotorsion, our values of 82.86° (SD: 4.6) right and 83.08° (SD: 5.2) left side shows that there seems to be a bony adaption toward a greater humeral retrotorsion.

### Limitations

However, it remains still unclear whether the posterior capsule thickening or the increased humeral retrotorsion accounts more for the obvious rotation range of motion limitation. As the number of enrolled athletes in this study due to the rather small number of high-level elite gymnasts training at a regional Olympic Training Centre is quite low the results have to be considered carefully. Although there was a significant correlation of higher age and the developing of GIRD compared to the control group this result for now is limited to the specific subgroup of athletes examined in this study. It might be possible that these findings are influenced by specific local or regional factors like performance selection, training methods, age of first participation in sport or prevention techniques.

## Conclusion

This study was able to identify a new type of GIRD in youth gymnasts and describes its age-related development and association with the sport-specific structural adaption of posterior capsular thickening and an increase in humeral retrotorsion in comparison to a control group of comparable age, sex, weight, and height. This bilateral type of GIRD shows no significant compensatory increase in external rotation range of motion, resulting in a decrease in total rotation range of motion and develops gradually according to age and training exposure. The study shows no association to periscapular muscle hypertrophy as a possible reason for the rotational range of motion limitation. Therefore, the design of potential intervention programs should consider the bilateral and symmetric load pattern. Following this, one focus for prevention exercises, therefore, could be bilateral stretching techniques for the posterior capsule and strengthening of the rotator cuff muscles.

## Data Availability

Data (e.g. study protocol, blinded measurement results, and statistical analysis plan) are available upon reasonable request by the corresponding author.

## Declarations

### Standards of reporting

Standards of reporting adhere to STROBE recommendations.
